# Short rare *hTERT*-VNTR2-2^nd ^alleles are associated with prostate cancer susceptibility and influence gene expression

**DOI:** 10.1186/1471-2407-10-393

**Published:** 2010-07-26

**Authors:** Se-Lyun Yoon, Se-Il Jung, Eun-Ju Do, Se-Ra Lee, Sang-Yeop Lee, In-Sun Chu, Wun-Jae Kim, Jaeil Jung, Choung Soo Kim, Sang-Hyeon Cheon, Sun-Hee Leem

**Affiliations:** 1 Department of Biology and Biomedical Science, Dong-A University, Busan, South Korea; 2 Department of Urology, College of Medicine, Dong-A University, Busan, South Korea; 3 Korean BioInformation Center, KRIBB, Daejeon, South Korea; 4 Department of Urology, College of Medicine, Chungbuk National University, Cheongju, South Korea; 5 Department of Urology, College of Medicine, Busan Paik Hospital, Inje University, Busan, South Korea; 6 Department of Urology, College of Medicine, Asan Medical Center, Ulsan University, Seoul, Korea; 7 Department of Urology, College of Medicine, Ulsan University Hospital, Ulsan University, Ulsan, South Korea

## Abstract

**Background:**

The *hTERT *(human telomerase reverse transcriptase) gene contains five variable number tandem repeats (VNTR) and previous studies have described polymorphisms for *hTERT*-VNTR2-2^nd^. We investigated how allelic variation in *hTERT*-VNTR2-2^nd ^may affect susceptibility to prostate cancer.

**Methods:**

A case-control study was performed using DNA from 421 cancer-free male controls and 329 patients with prostate cancer. In addition, to determine whether the VNTR polymorphisms have a functional consequence, we examined the transcriptional levels of a reporter gene linked to these VNTRs and driven by the *hTERT *promoter in cell lines.

**Results:**

Three new rare alleles were detected from this study, two of which were identified only in cancer subjects. A statistically significant association between rare *hTERT*-VNTR2-2^nd ^alleles and risk of prostate cancer was observed [OR, 5.17; 95% confidence interval (CI), 1.09-24.43; *P *= 0.021]. Furthermore, the results indicated that these VNTRs inserted in the enhancer region could influence the expression of *hTERT *in prostate cancer cell lines.

**Conclusions:**

This is the first study to report that rare *hTERT *VNTRs are associated with prostate cancer predisposition and that the VNTRs can induce enhanced levels of *hTERT *promoter activity in prostate cancer cell lines. Thus, the *hTERT*-VNTR2-2^nd ^locus may function as a modifier of prostate cancer risk by affecting gene expression.

## Background

Telomeres play a critical role in the maintenance of genomic stability in all eukaryotes [1]. Telomerase, a ribonucleoprotein complex with RNA template and catalytic subunit *hTERT *core components, is able to add reiterated telomeric repeat sequences to the very ends of chromosomes. While the telomerase RNA template is present in almost all human cells, *hTERT *was found to be rate limiting for telomerase activity [2,3]. Telomerase expression is confined primarily to the germ line and regenerating tissues in adults [3,4]. Activation of telomerase to maintain telomeres is required for self-renewal and proliferative expansion of a number of cell types, including stem cells, activated lymphocytes and cancerous cells [2,3,5]. Human telomerase is highly active in more than 85% of primary cancers, but not in most differentiated somatic tissues. Ectopic expression of *hTERT *in otherwise mortal human cells induces efficient elongation of telomeres and permanent cell growth [2,3,5].

In our previous work, we described the isolation and characterization of the complete genomic human telomerase gene [6]. The *hTERT *gene contains four blocks of polymorphic minisatellite regions: two blocks of VNTRs within intron 2 (VNTR2-1^st ^and VNTR2-2^nd^) and two within intron 6 (VNTR6-1^st ^and VNTR6-2^nd^) [6,7]. In a previous study, we found that *hTERT*-VNTR2-2^nd ^contains minisatellites with 61-bp periodicity. We identified four alleles of *hTERT*-VNTR2-2^nd ^ranging from 40 to 44 copies of the 61-bp repeat unit, with 44 the most common, and a corresponding degree of heterozygosity of 0.476 [6]. The repeats diverge by approximately 10%. Analysis of segregation of the minisatellites in families revealed that all four VNTR alleles are transmitted in a Mendelian inheritance pattern [6].

Despite the lack of understanding about the function of repetitive DNA, these elements have been implicated in the pathogenesis of human genetic disease [8-11]. VNTR polymorphisms have also been reported to affect gene expression [12,13]. The high level of VNTR polymorphism and consequent heterozygosity pose a problem as to what haplotypes are present in a given cell and which of them is responsible for gene expression. Comparison of normal and cancer tissue from patients revealed rearrangements of at least two VNTR regions of *hTERT*, suggesting that the minisatellites might be associated with activation of telomerase expression in cancer cells [6]. Many repeats of *hTERT*-VNTR2-1^st ^include the canonical CACGTG binding site of the MYC family of oncogenic transcription factors, that is also present in the promoter region; overexpression of c-MYC activates *hTERT *transcription through this promoter element [6,14].

In this study, we investigated how allelic variation in *hTERT*-VNTR2-2^nd ^affects susceptibility to sporadic cancers of the prostate. A case-control study using a PCR-based method was performed to compare the allelic distribution of *hTERT*-VNTR2-2^nd ^in DNA samples from cancer-free controls and patients with cancer. We identified three novel minisatellite alleles in the *hTERT*-VNTR2-2^nd ^region. Furthermore, to determine if the VNTR polymorphisms are functional, we examined the transcriptional levels of a *hTERT *promoter-driven reporter gene in the presence of VNTRs with a varying number of repeats in prostate cancer cell lines. Our results indicate that allelic variations in the minisatellites of *hTERT*-VNTR2-2^nd ^may be related to susceptibility to prostate cancer by affecting the expression level of *hTERT *in cancer cell lines.

## Methods

### Study population

The degree of minisatellite polymorphism in *hTERT-*VNTR2-2^nd ^was assessed in 421 unrelated healthy adult males. In addition, we performed a case-control study in which we compared DNA from these 421 cancer-free controls to samples obtained from 329 patients with prostate cancer. Controls were selected from the Department of Preventive Medicine and Internal Medicine of Dong-A University hospitals between 2000 and 2004 (Busan, Korea). A total of 421 male individuals in the control group who had no personal history of cancers or current cancer were recruited and completed an interview. The controls had a similar age distribution to the cancer patients (control mean age, 66.7 y, range 50-89 y; patients mean age, 67.9 y, range 50-88 y). Subjects were recruited from five different hospitals in four different cities in Korea: Dong-A University Hospital and Busan Paik Hospital in Busan, Chungbuk National University Hospital in Cheongju, Asan Medical Center in Seoul, and Ulsan University Hospital in Ulsan. Prior to collection, each participating subject provided his informed consent. The Committees of Bioethics of Dong-A University [#IRB-06-10-02 & IRB-07-10-7; Busan, Korea] and Chungbuk National University Hospital [#IRB-2006-1; Cheongju, Korea] approved the study design and procedures.

### PCR analysis of *hTERT*-VNTR2-2^nd^

To assess the degree of polymorphism of *hTERT*-VNTR2-2^nd^, we analyzed genomic DNA samples from 329 prostate cancer patients and 421 healthy, unrelated adult males. For PCR experiments, genomic DNA from peripheral blood lymphocytes was extracted as described [10]. Primer sequences used in this study are as follows: *hTERT*-VNTR2-2^nd ^F-TGGGAGCATCACTCACAGGA and *hTERT*-VNTR2-2^nd ^R-GGAACACAGCCAACCCCTTA [6]. PCR analysis of human DNA samples was performed using the Promega *Go Taq *polymerase (Promega, WI) with 100 ng genomic DNA. Genomic DNA was amplified using primers under the following standard PCR conditions: 50 mM KCl; 10 mM Tris-HCl, pH 9.0; 3 mM MgCl_2_; and 0.2 mM dTTP, dCTP, dGTP, and dATP in a final volume of 40 μl. PCR was conducted in a 9700 Thermalcycler (Perkin-Elmer, CA, USA) with cycle conditions consisting of 94°C for 2 min, 30 cycles of 45 sec at 94°C and 69°C for 2 min 30 sec. The last elongation step was extended to 7 min at 72°C. PCR products were analyzed by gel electrophoresis (1 volt/cm) in TAE buffer through 0.8% agarose gel.

**Figure 1:**
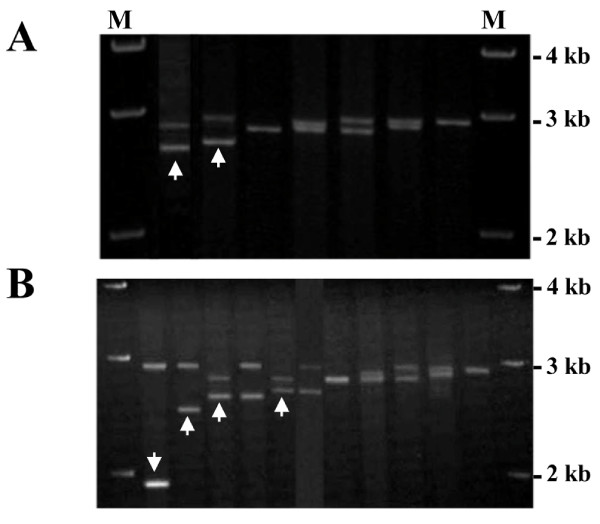
**Allele typing at *hTERT*-VNTR2-2^nd ^in cancer-free controls and patients with prostate cancer**. (A) Electrophoretic patterns of PCR products of *hTERT*-VNTR2-2^nd ^in controls. Four *hTERT*-VNTR2-2^nd ^alleles and six haplotype patterns were detected in DNA from 421 cancer-free male controls. One new rare allele (39 copies of the repeat unit) and one previously reported rare allele (40 copies) identified in the control group are indicated with arrows. (B) Electrophoretic patterns of PCR products of *hTERT*-VNTR2-2^nd ^in patients with prostate cancer. Seven *hTERT*-VNTR2-2^nd ^alleles and eleven haplotype patterns were detected in DNA from 329 patients with prostate cancer. The three new rare alleles (28, 37, and 39 copies) and one previously reported rare allele (40 copies) identified in prostate patients are indicated with arrows. The 28- and 37-copy alleles were detected only in patients with prostate cancer. The first and last lanes correspond to a 1-kb marker (M).

Three newly identified *hTERT*-VNTR2-2^nd ^alleles were amplified from human genomic DNA using a PCR method for sequencing (Figure 1). After electrophoresis, the PCR products were extracted using a gel extraction kit (Qiagen, CA), and the repeat sequence of the PCR product was analyzed.

### Plasmid construction

**Figure 2:**
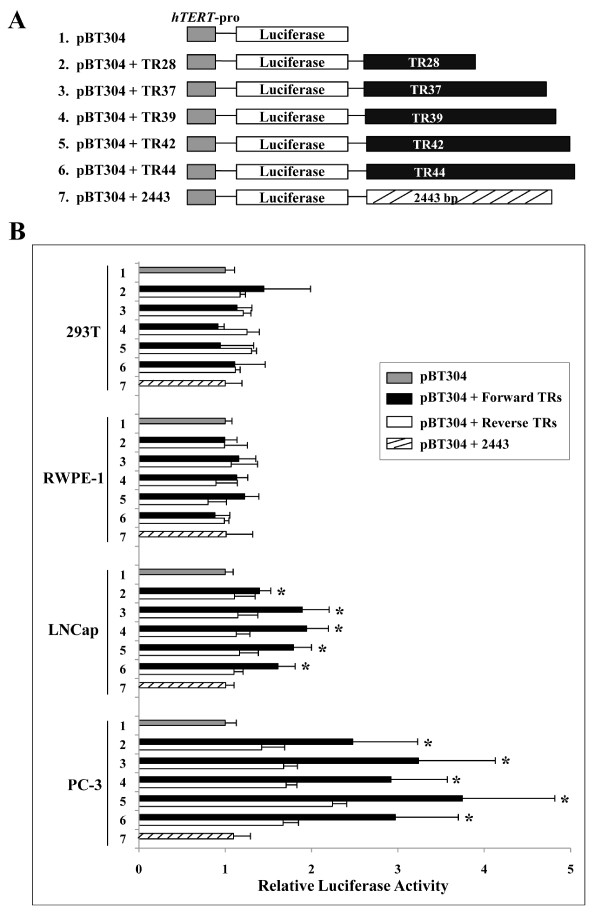
**Effect of allelic types of *hTERT*-VNTR2-2^nd ^in *hTERT*-promoter luciferase constructs**. (A) The structure of the pBT304 (#1) and TR reporter constructs. The gray square indicates the *hTERT *promoter region. The open square represents the open reading frame of luciferase. The black squares include the VNTR polymorphic regions of *hTERT*-VNTR2-2^nd^. Five different sizes of TR (28-44 copies) were inserted in the pBT304 plasmid: pBT304 + TR28, pBT304 + TR37, pBT304 + TR39, pBT304 + TR42, and pBT304 + TR44 (#2-6). All TR-containing plasmids were constructed in both the forward and reverse directions of the reporter gene. The pBT304+2443 plasmid (#7) was made by insertion of an irrelevant 2443-bp fragment instead of TR. (B) The effects of VNTR polymorphism on *hTERT *gene expression in the luciferase reporter system. Four different cell lines (293T, embryonic kidney, RWPE-1, prostate, LNCap and PC-3, prostate cancer) were transfected with 12 different plasmids [#1-7, with paired forward- and reverse-TR-containing constructs (#2-6)]. * denotes statistical significance (*P *< 0.05).

A fragment of the *hTERT *promoter (-304 to +40) [15] was amplified from a bacterial artificial chromosome clone containing the *hTERT *genomic sequence [6] and inserted into *Kpn*I/*Nhe*I sites of the luciferase reporter vector pGL3-Basic (Promega) to generate the pBT304 construct (Figure 2-A). Two common alleles (TR42, TR44) and three rare alleles (TR28, TR37, TR39) were amplified from genomic DNA from patients with prostate cancer (Figure 1-B) and inserted into the *Bgl*II/*Sal*I sites of pBT304 to make the reporter plasmids pBT304+TR28, pBT304+TR37, pBT304+TR39, pBT304+TR42, and pBT304+TR44, respectively (Figure 2-A). All plasmids containing TR were also constructed in the reverse orientation in the reporter to examine the effect of TR orientation on gene expression. Moreover, to compare the effect of similarly sized irrelevant DNA sequences on luciferase expression, the pBT304+2443 plasmid was made by insertion of a 2443-bp fragment (UCSC, >hg18_dna; Chr5: 4359328-4361770) into the *Bgl*II/*Sal*I sites instead of TR. All constructs were confirmed by DNA sequencing.

### Cells and luciferase assay

The following human cell lines were examined for the effect of VNTRs on *hTERT *expression: 293T/HEK293T (human embryonic kidney cell line obtained from the Korean Cell Line Bank (KCLB), South Korea), RWPE-1 (prostate cell line obtained from the American Type Culture Collection (ATCC), USA), PC-3 (prostate cancer cell line obtained from KCLB), and LNCap (prostate cancer cell line obtained from KCLB). For the luciferase assay, cells (1 × 10^5^) were seeded in 12-well plates, cultured overnight, and transfected with the *hTERT *promoter-luciferase plasmids (0.5 μg per well) by using the FuGENE6 transfection reagent (Roche Diagnostics, USA) at a ratio of DNA to FuGENE6 of 1:3. The cells were analyzed using a dual-luciferase reporter assay system (Promega) 48 h after completion of the transfection procedure. *Firefly *luciferase activity was normalized according to *Renilla *luciferase activity and expressed as relative luciferase units to reflect promoter activity. Triplicate transfections of each construct were examined for each experiment, and final results were calculated from four independent experiments.

### Statistical Analysis

The degree of polymorphism, which ranges from 0 to 1, generally increases with the number of alleles. To evaluate the probability of two randomly chosen alleles being different (heterozygosity) at a given locus, a measure of genetic diversity was calculated using the method described by Chakravarti and Lynn [16]. Regression analyses were performed to determine the odds ratios (ORs) of associations between control and patient groups. ORs were estimated using the natural logarithm and its standard error. Where relevant, we used a chi-squared test with one degree of freedom to assess statistical significance. Differences were considered significant for confidence intervals (CIs) of 95%. All tests were two-sided, with *P *< 0.05 considered statistically significant. Statistical analyses were performed using MS Excel with CHITEST and R statistical software (v2.5.1, http://www.r-project.org) with chisq.test for the calculation of chi-squared values.

## Results

### Three new rare alleles at *hTERT*-VNTR2-2^nd^

In this study, we analyzed the degree of polymorphism within the *hTERT*-VNTR2-2^nd ^repeats by PCR amplification of human genomic DNA samples isolated from 421 cancer-free males and 329 prostate cancer patients (Figure 1). We assessed the degree of heterozygosity in each sample group according to allelic distribution. The heterozygosity of *hTERT*-VNTR2-2^nd ^was 0.4782 in controls and 0.4964 in prostate cancer patients, with no statistically significant difference between the two groups. We identified 5 alleles in 421 control samples ranging from 39 to 44 copies of the 61-bp repeat unit, with 44 copies present in the most common allele (Table 1). Compared to a previous report [6], we observed one novel allele, with 39 copies of the 61-bp repeat unit, in the control group (Figure 1-A). We also observed two additional novel alleles, of 28 and 37 copies, in the prostate cancer group (Figure 1-B). Thus, three new alleles were identified at the *hTERT-*VNTR2-2^nd ^locus in this study: one in controls and two cancer-specific alleles in prostate cancer patients (Table 1). The genotype distribution of *hTERT*-VNTR2-2^nd ^variants in prostate cancer patients and control subjects is shown in Table 2. Eleven patterns were identified in prostate cancer patients; only six patterns were observed in controls (Figures 1-A and 1-B). Five rare cancer-specific genotypes were identified in patients with prostate cancer (28/44, 37/44, 39/44, 40/42 and 40/44) (Figure 1-B and Table 2).

**Table 1:** Haploid types of *hTERT*-VNTR2-2^nd ^in male controls and patients with prostate cancer

Haplotype		Male controls	Prostate cancer	OR (95% CI)	*P*
		
TR	Size (bp)	842 [N(%)]	658 [N(%)]		
28	1934	-	1 (0.0015)	-	0.258
37	2483	-	1 (0.0015)		0.258
39	2605	1 (0.0012)	4 (0.0061)	5.14 (0.57-46.13)	0.103
40	2666	1 (0.0012)	2 (0.0030)	2.56 (0.23-28.34)	0.426
42	2788	307 (0.3646)	232 (0.3526)	0.95 (0.77-1.17)	0.630
43	2849	21 (0.0249)	13 (0.0198)	0.79 (0.39-1.59)	0.503
44	2910	512 (0.6081)	405 (0.6155)	1.03 (0.84-1.27)	0.770

**Table 2:** Allelic genotypes and frequency in male controls and cases

Genotype	Male controls 421 [N(%)]	Prostate cancer 329 [N(%)]	OR (95% CI)	*P*
**28**/44	0 (0.00)	1 (0.30)	-	0.2576
**37**/44	0 (0.00)	1 (0.30)	-	0.2576
**39**/42	1 (0.24)	1 (0.30)	1.28 (0.08-20.5)	0.8611
**39**/44	0 (0.00)	3 (0.91)	-	0.0496*
**40**/42	0 (0.00)	1 (0.30)	-	0.2576
**40**/44	1 (0.24)	1 (0.30)	1.28 (0.08-20.5)	0.8611

42/42	54 (12.83)	37 (11.25)	0.86 (0.55-1.34)	0.5107
42/43	6 (1.43)	6 (1.82)	1.28 (0.41-4.02)	0.6660
42/44	192 (45.61)	150 (45.59)	1.00 (0.75-1.34)	0.9972
43/44	15 (3.56)	7 (2.13)	0.59 (0.24-1.46)	0.2477
44/44	152 (36.10)	121 (36.79)	1.03 (.076-1.39)	0.8491

**C/C**	419 (99.52)	321 (97.57)	5.22 (1.10-24.76)	
**C/R**	2 (0.48)	8 (2.43)	*P *= 0.02*	

Bold numbers represent the rare alleles of *hTERT*-VNTR2-2^nd^. C, common alleles; R, rare alleles. * Statistically significant (*P *< 0.05)

### Association of rare *hTERT*-VNTR2-2^nd ^alleles with prostate cancer

For further analysis, *hTERT*-VNTR2-2^nd ^alleles were grouped into two sets (common and rare alleles), according to their frequency in the control population. The expected frequency for rare alleles was considered ≤1% in this study. Analysis of the genotypes found in individual cancer patients and healthy controls revealed that having a rare allele (2.43% of patients, 0.48% of controls) was associated with relative prostate cancer odds of 5.22 (CI: 1.10-24.76; *P *= 0.02) (Table 2). Furthermore, the repeat sizes of all rare alleles (28, 37, 39 and 40 copies) were shorter than common alleles (42, 43, and 44 copies). Therefore, there is a statistically significant risk for developing prostate cancer associated with the presence of shorter, rare alleles. Table 3 summarizes the frequency of rare and common *hTERT*-VNTR2-2^nd ^alleles (haplotype) among cancer patients and controls. Interestingly, in patients with prostate cancer, the total rate of rare *hTERT*-VNTR2-2^nd ^alleles was 1.22%, compared to 0.24% in cancer-free male controls. Analysis of these data revealed a statistically significant association between rare *hTERT*-VNTR2-2^nd ^alleles and risk of prostate cancer (OR, 5.17; 95% CI, 1.09-24.43; *P *= 0.021) (Table 3).

**Table 3:** The rare *hTERT *-VNTR2-2^nd ^alleles associated with prostate cancer

Samples	No. of alleles	Common alleles				Rare alleles					OR (95% CI)	*P*
				
		42	43	44	Total	28	37	39	40	Total		
Male controls	842 (%)	307 (36.46)	21 (2.49)	512 (60.81)	840 (99.76)	0	0	1	1	2 (0.24)	1 (reference)	-
Prostate Cancer	658 (%)	232 (35.26)	13 (1.97)	405 (61.55)	650 (98.78)	1	1	4	2	8 (1.22)	5.17 (1.09-24.43)	0.021*

* Statistically significant (*P *< 0.05)

We further analyzed the *hTERT*-VNTR2-2^nd ^allele types on the basis of subject age. Table 4 shows the genotype distribution in patients according to age at diagnosis and in controls according to age at the time of study enrollment. In the control group, we found that there was no difference in the frequencies of short rare alleles between younger (<65 years) and older (≥65 years) individuals (*P *= 0.21). In comparison to older patients (≥65 years), however, we found that younger patients (<65 years) had an increased ratio (3.79, CI: 0.89-16.18; *P *= 0.05) of association between rare *hTERT*-VNTR2-2^nd ^alleles and prostate cancer. The frequency of rare alleles was higher in younger cases (4.9%) than in older cases (1.3%). Specifically, a comparison between controls and cancer patients by age group demonstrated a statistically significant difference (*P *= 0.02) in the association ratio between prostate cancer and rare *hTERT*-VNTR2-2^nd ^alleles in younger patients (Table 4).

**Table 4:** Frequency of *hTERT*-VNTR2-2^nd ^and risk of prostate cancer by age

	Male controls		Prostate cancer cases		OR (95% CI); *P*
**Age at diagnosis**	**Total cases**	**Rare alleles**	**Total cases**	**Rare alleles**	**Reference** **(Controls of the same age)**

Younger (<65 years)	185	0 (0%)	103	5 (4.9%)	ND; *P *= 0.002*
Older (≥65 years)	236	2 (0.9%)	226	3 (1.3%)	1.57 (0.26-9.46); *P *= 0.62

Reference (Older group)	ND; *P *= 0.21		3.79 (0.89-16.03); *P *= 0.05		

* Statistically significant (*P *< 0.05)

We used additional clinicopathological information collected from 2005-2006 at the Asan Medical Center in Korea [17,18] and in 2006 at the National Cancer Center in Korea [19] (Table 5) to further characterize prostate cancer patients. Tumor, node, and metastases (TNM) stage and histopathological classifications were analyzed according to the World Health Organization (WHO) system. Prostate cancer was evaluated using TNM stage, Gleason score, prostate-specific antigen (PSA), Jewett-Whitmore system, and the Tumor grade (Table 5). Prostate tumors were grouped according to their classification, and we then estimated the frequency of each stage in the total cancer group and in the rare allele group by Pearson's chi-squared test (Table 5).

**Table 5:** Tumor characteristics in cases with total cases and rare alleles

Characteristic	2006 AMC*	2006 NCC**	2005 AMC*	This study	Rare alleles	**χ**^**2**^-value*** (*P *value)
Age						
Mean	63.9 (42-78)	64.1 (46-80)	64.3 (40-83)	67.9 (50-88)	64.7 (55-81)	
T-stage						
T1	0 (0.0%)	0 (0.0%)	130 (41.0%)	42 (13.2%)	0 (0.0%)	6.0132 (df = 3)
T2	116 (62.4%)	52 (63.4%)	183 (57.7%)	139 (43.8%)	3 (42.9%)	*P *= 0.1110
T3	68 (36.6%)	29 (35.4%)	4 (1.3%)	95 (30.0%)	1 (14.3%)	
T4	2 (1.1%)	1 (1.2%)	0 (0.0%)	41 (12.9%)	3 (42.9%)	
N-stage						
N0	182 (97.8%)	45 (91.8%)	291 (91.8%)	265 (85.8%)	3 (42.9%)	6.7334 (df = 1)
N1	4 (2.2%)	4 (8.2%)	26 (8.2%)	44 (14.2%)	4 (57.1%)	*P *= 0.0095
M-stage						
M0	158 (84.9%)	45 (91.8%)	280 (88.3%)	247 (75.5%)	3 (37.5%)	4.1265 (df = 1)
M1	28 (15.1%)	4 (8.2%)	37 (11.7%)	80 (24.5%)	5 (62.5%)	*P *= 0.0422
Gleason score						
2-6	34 (18.3%)	22 (31.9%)	126 (39.8%)	71 (23.5%)	1 (12.5%)	2.839 (df = 2)
7	108 (58.0%)	33 (47.8%)	98 (30.9%)	95 (31.5%)	1 (12.5%)	*P *= 0.2418
8-10	44 (23.7%)	14 (20.3%)	93 (29.3%)	136 (45.0%)	6 (75.0%)	
PSA (ng/ml)						
<10	110 (59.1%)	41 (49.4%)	142 (44.8%)	29 (34.1%)	0 (0.0%)	2.253 (df = 2)
10-20	57 (30.7%)	21 (25.3%)	102 (32.2%)	8 (9.4%)	0 (0.0%)	*P *= 0.3241
>20	19 (10.2%)	21 (25.3%)	73 (23.0%)	48 (56.5%)	3 (100%)	
Jewett-Whitmore system						
A				35 (10.7%)	0 (0.0%)	7.3884 (df = 3)
B	ND	ND	ND	126 (38.4%)	1 (12.5%)	*P *= 0.0605
C				67 (20.4%)	1 (12.5%)	
D				100 (30.5%)	6 (75.0%)	
Tumor grade						
Well			25 (7.9%)	9 (3.0%)	0 (0.0%)	0.6087 (df = 2)
Moderate	ND	ND	101 (31.9%)	62 (20.5%)	1 (12.5%)	*P *= 0.7376
Poor			191 (60.3%)	231 (76.5%)	7 (87.5%)	

*Data of Asan Medical Center, Korea; ** Data of National Cancer Center, Korea; *** Pearson's Chi-squared test was performed between total cases with prostate cancer and cases with rare alleles used in this study.

We determined the proportion of TNM stages within the short rare alleles group and the prostate cancer group. The frequency of short rare allele patients in the T4 stage was slightly higher than in the total prostate cancer group (*P *= 0.1110), and the frequency of short rare allele patients in the N1 and M1 stages was significantly higher than in the total prostate cancer group (*P *= 0.0146 and *P *= 0.0422, respectively). We then classified patients into three different ranges of PSA, a biomarker for detecting prostate cancer [20], to determine the relationship between PSA level and short rare alleles in the prostate cancer group. Although three of the eight patients with rare alleles showed high levels of PSA (>20 ng/ml), no statistically significant differences were observed due to the small group size. According to the Jewett-Whitmore system, prostate cancer is classified according to anatomical view and spread. A and B are early stages; stage C and D cancer invades most of the prostate and spreads to other organs/tissues [21]. In the present study, 10.7% of patients were classified as group A, 38.4% as group B, 20.4% as group C, and 30.5% as group D. Prostate cancer patients with rare alleles were distributed as follows: A (0%), B (12.5%), C (12.5%), and D (75%). The frequency of patients with a rare allele in the 'D' group was higher than in the total prostate cancer group, but, as with T4 staging and PSA level, no statistically significant difference was observed (*P *= 0.0605) in the Jewett-Whitmore system.

### Effect of VNTR polymorphisms on *hTERT *expression

To determine if VNTR polymorphisms affect *hTERT *expression levels, we constructed reporter vectors that contained the *hTERT *promoter, luciferase gene, and one of five differently sized VNTR repeats ranging from 28 to 44 copies of the 61-bp tandem repeat region of *hTERT*-VNTR2-2^nd ^(PCR product sizes: approximately 1,930-2,900 bp) inserted in the enhancer region of the pGL3-Basic vector (Figure 2-A). The effects of these VNTRs in cell lines were examined by transfection of the luciferase plasmid pBT304 with or without each VNTR repeat variant. In 293T (human embryonic kidney cell line) and RWPE-1 (human prostate cell line) cells, no statistically significant differences in luciferase activity were observed between controls (pBT304 and pBT304 + 2443) and VNTR-containing cells (pBT304 + TR, with both orientations of five different TRs) (Figure 2-B). Interestingly, when we transfected these plasmids into two different prostate cancer cell lines (PC3 and LNCap cells), luciferase assays revealed that forward-VNTRs stimulated the activity of the *hTERT *promoter, leading to approximately 1.4 to 1.9-fold increases in relative luciferase activity in LNCap cells and 2.5 to 3.7-fold increases in PC3 cells (Figure 2-B). Notably, the PC3 cell line demonstrated a greater increase in luciferase activity as compared to the LNCap cell line. However, when we recalculated relative luciferase activity by comparing the forward- and reverse-VNTRs of each variant, the increase in luciferase activity was similar in both cell lines, ranging from 1.4 to 1.7-fold in LNCap cells and 1.7 to 1.9-fold in PC3 cells. Similarly sized irrelevant DNA sequences (pBT304 + 2443) had no effect on luciferase expression.

## Discussion

A characteristic of tandem repetitive sequences is their ability to give rise to variants that contain increased or decreased numbers of the repeat unit. A recent study of genetic factors involved in susceptibility to insulin-dependent diabetes mellitus demonstrates the importance of population-based association studies in understanding the effects of VNTRs [22]. Some minisatellite alleles are associated with human disorders and with differential expression of a nearby gene [23], which lends support to the concept that variations in a minisatellite locus may have biological significance. Case-control studies have implicated rare *HRAS *minisatellite alleles in cancer risk [24,25], and specific *HRAS *minisatellite alleles have demonstrated enhancer or suppressor activity *in vitro *[26]. VNTRs in introns may also affect mRNA splicing [27]. In addition, rare VNTR alleles have been associated with a higher risk of various types of cancer [10,23,28]. These findings suggest a potential biological role for rare minisatellite alleles in cancer predisposition; however, the nature of the phenomena underlying the association between minisatellites and cancer has remained unclear.

Telomerase activity is detected in approximately 85% of malignant tumor cells, and its presence can be used to diagnose cancer [29]. Regulation of the *hTERT *gene is a major factor in telomerase activity [30], and the expression level of *hTERT *is highly correlated with telomerase activity in normal and cancer cells [31]. *hTERT *includes four polymorphic minisatellites, 42- and 61-bp minisatellites in intron 2 (VNTR2-1^st ^and 2-2^nd^) and 38- and 36-bp minisatellites in intron 6 (VNTR6-1^st ^and 6-2^nd^), as well as an apparently monomorphic 28-bp minisatellite in intron 12 (TR12) [6]. The influence of these minisatellites on gene expression is not known, but they may intervene in regulatory mechanisms affecting *hTERT *expression. The DNA from cancer tissues of one patient with a kidney tumor had a simultaneous rearrangement of *hTERT*-VNTR6-1^st ^and 6-2^nd ^[6,7], an observation that suggests that chromosomal rearrangements involving these VNTRs may be associated with the activation of telomerase expression in cancer. Moreover, according to reports by Wang *et al*. [23], MNS16A, which is located downstream of *hTERT*, was reported to have an effect on *hTERT *expression and telomerase activity. In a previous study, we characterized *hTERT*-VNTR2-2^nd ^as having four alleles containing a different number of the repeat unit in 103 normal, unrelated individuals [6], and we found in a pilot study that *hTERT*-VNTR2-2^nd ^regulated *hTERT *promoter activity in a reporter gene assay.

We hypothesized that *hTERT*-VNTR2-2^nd ^polymorphisms could play a role in activating *hTERT *during tumorigenesis. The present case-control study was performed on DNA from 421 cancer-free male controls and patients with 329 prostate cancers using PCR methods to determine the frequency of rare alleles. These data suggest a statistically significant (*P *= 0.021) increased incidence of rare *hTERT*-VNTR2-2^nd ^alleles in prostate cancer patients (1.22%) compared to male controls (0.24%). Moreover, the frequency of rare alleles among prostate cancer patients was significantly higher in younger patients (4.9%) than in older patients (1.3%) (*P *= 0.02). These results suggest that rare *hTERT*-VNTR2-2^nd ^alleles may be genetically associated with prostate cancer.

Prostate cancer is often diagnosed at an advanced stage, and metastatic prostate cancer is incurable. Although the advent of the PSA test and increased public awareness have improved early detection rates, a large number of patients still die of metastatic prostate cancer, reflecting the presence of occult metastatic disease [32]. The diagnosis of early-stage prostate cancer is imperative for its successful treatment, but the methods employed in diagnosis suffer from significant limitations. Here, we found a different proportion of TNM stages between the short rare-allele group and the prostate cancer group as a whole. Prostate cancer tissue classified as M1-stage, N1-stage, or Jewett-Whitmore group 'D' is associated with a poor prognosis. Using the Jewett-Whitmore system, the frequency of cases with a rare allele in the 'D' group was higher than in the total prostate cancer group, but no statistically significant difference was observed due to the limited number of samples. However, there were significant relationships between the short rare allele and the appearance of regional lymph nodes (N1 stage) and metastasis (M1 stage). These results suggest that prostate cancer patients with rare *hTERT*-VNTR2-2^nd ^alleles have a poorer prognosis than patients with common alleles.

This study is the first to calculate the risk of prostate cancer attributable to rare *hTERT*-VNTR2-2^nd ^alleles and report that rare *hTERT*-VNTR2-2^nd ^alleles of the human telomerase gene contribute to prostate cancer predisposition in the general population. This finding may prove useful as a diagnostic biomarker of increased risk for prostate cancer and cancer progression, though the short rare alleles group is too small to be common in prostate cancer cases. Further research is necessary to identify the specific minisatellite variants that confer predisposition and to further evaluate the associations between *hTERT*-VNTR2-2^nd ^alleles and cancers of the prostate.

Sequence analyses highlighted possible mechanisms underlying the relationship we observed between *hTERT*-VNTR2-2^nd ^alleles and prostate cancer. When the minisatellite sequences were analyzed using the Transfac software (MATCH™ public version 1.0; http://www.gene-regulation.com/pub/databases.html), we found five putative binding sites for the transcription factor, estrogen receptor 1 (ER1), in the repeat region of *hTERT*-VNTR2-2^nd^. The estrogen receptor α subunit is expressed in androgen receptor-dependent prostate cancer, suggesting that the ER pathway may be involved in prostate cancer. We also found binding sites in repeat regions for the transcription factors v-ErbA (5 sites) and NF-kappaB (3 sites), which are related to cancer development [33-35]. However, these latter sites were found by scanning all of the putative regulatory elements (minisatellites, etc.), including those in introns. In a report by Brandt et al. [36], a transcriptional regulation mechanism has been described that depends on the length of a CA repeat in intron 1 (CA simple sequence repeat 1) of the EGFR gene. This mechanism may also be involved in regulation of the *hTERT *gene, with oncogenic proteins binding to sites in repeat regions and stimulating *hTERT *expression during cancer progression.

On the basis of these putative binding sites, we investigated the effects of VNTR polymorphism on *hTERT *gene expression in cell lines by using the luciferase reporter system to observe the effect of VNTRs on the *hTERT *promoter. In PC3 and LNCap prostate cancer cell lines, all five VNTR constructs enhanced the activity of the *hTERT *promoter, but with no clear relationship between the number of repeats and the change in expression. This finding may be indicative of the limitations of *in vivo *transient transcriptional regulation assays. Alternatively, the high density of putative binding sites within the repeat array for transcription factors such as ER, v-ErbA and NF-kappaB may enhance *hTERT *gene expression independently of the number of tandem repeats. When we examined the luciferase activity of the *hTERT *promoter that included the VNTRs in gastric cancer, no increase was detected (data not shown). Therefore, the high density of putative binding sites within the repeat array for transcription factors such as ER may enhance *hTERT *gene expression with cell line specificity.

## Conclusions

Our findings suggest that non-coding regions such as minisatellite repeat regions may influence gene activity. Further investigations of the epidemiological associations between minisatellites and cancer, and the role of repeat regions in gene regulation, are warranted. This study should also provide a helpful reference for understanding the possible function of minisatellites and the complex genomic properties of genes.

## Abbreviations

*hTERT*: (human telomerase reverse transcriptase); VNTR: (variable number tandem repeats); CI: (confidence interval); ORS: (odds ratios); ER1: (estrogen receptor 1).

## Competing interests

The authors declare that they have no competing interests.

## Authors' contributions

SLY conceived of the study and carried out the PCR amplification, vector construction and luciferase assay. EJD and SRL performed sample selection, genomic DNA preparation and PCR amplification. SYL and ISC carried out the statistical analyses, bioinformatic analysis and contributed to the interpretation of the results. SIJ, WJK, JIJ, CSK and SHC collected the samples, investigated the clinical records of the patients and contributed to the interpretation of the results. SHL designed the study concept, interpreted the results and drafted the manuscript. Other: All authors approved the version to be published. SHL is guarantor.

## Authors' information

^1^Department of Biology and Biomedical Science, Dong-A University, Busan, South Korea. ^2^Department of Urology, College of Medicine, Dong-A University, Busan, South Korea. ^3^Korean BioInformation Center, KRIBB, Daejeon, South Korea. ^4^Department of Urology, College of Medicine, Chungbuk National University, Cheongju, South Korea. ^5^Department of Urology, College of Medicine, Busan Paik Hospital, Inje University, Busan, South Korea. ^6^Department of Urology, College of Medicine, Asan Medical Center, Ulsan University, Seoul Korea. ^7^Department of Urology, College of Medicine, Ulsan University Hospital, Ulsan University, Ulsan, South Korea.

## Pre-publication history

The pre-publication history for this paper can be accessed here:

http://www.biomedcentral.com/1471-2407/10/393/prepub

## References

[B1] Chiu CP Harley CB Replicative senescence and cell immortality: the role of telomeres and telomerase Proc Soc Exp Biol Med 1997 214 2 99 106 9034126 10.3181/00379727-214-44075

[B2] Shay JW Wright WE Mutant dyskerin ends relationship with telomerase Science 1999 286 5448 2284 2285 10.1126/science.286.5448.2284 10636790

[B3] Yoshida R Kiyozuka Y Ichiyoshi H Senzaki H Takada H Hioki K Tsubura A Change in telomerase activity during human colorectal carcinogenesis Anticancer Res 1999 19 3B 2167 2172 10472326

[B4] Kim NW Piatyszek MA Prowse KR Harley CB West MD Ho PL Coviello GM Wright WE Weinrich SL Shay JW Specific association of human telomerase activity with immortal cells and cancer Science 1994 266 5193 2011 2015 10.1126/science.7605428 7605428

[B5] Cerni C Telomeres, telomerase, and myc. An update Mutat Res 2000 462 1 31 47 10.1016/S1383-5742(99)00091-5 10648922

[B6] Leem SH Londoño-Vallejo JA Kim JH Bui H Tubacher E Solomon G Park JE Horikawa I Kouprina N Barrett JC Larionov V The human telomerase gene: complete genomic sequence and analysis of tandem repeat polymorphisms in intronic regions Oncogene 2002 21 5 769 777 10.1038/sj.onc.1205122 11850805

[B7] Szutorisz H Palmqvist R Roos G Stenling R Schorderet DF Reddel R Lingner J Nabholz M Rearrangements of minisatellites in the human telomerase reverse transcriptase gene are not correlated with its expression in colon carcinomas Oncogene 2001 20 20 2600 2605 10.1038/sj.onc.1204346 11420670

[B8] Krontiris TG Devlin B Karp DD Robert NJ Risch N An association between the risk of cancer and mutations in the HRAS1 minisatellite locus N Engl J Med 1993 329 8 517 523 10.1056/NEJM199308193290801 8336750

[B9] Bennett ST Lucassen AM Gough SCL Powell EE Undlien DE Pritchard LE Merriman ME Kawaguchi Y Dronsfield MJ Pociot F Nerup J Bouzekri N Cambon-Thomsen A Rønningen KS Barnett AH Bain SC Todd JA Susceptibility to human type I diabetes at IDDM2 is determined by tandem repeat variation at the insulin gene minisatellite locus Nat Genet 1995 9 3 284 292 10.1038/ng0395-284 7773291

[B10] Jeong YH Kim MC Ahn EK Seol SY Do EJ Choi HJ Chu IS Kim WJ Kim WJ Sunwoo Y Leem SH Rare exonic minisatellite alleles in MUC2 influence susceptibility to gastric carcinoma PLoS ONE 2007 2 11 e1163 10.1371/journal.pone.0001163 18000536 PMC2065792

[B11] Seol SY Lee SY Kim YD Do EJ Kwon JA Kim SI Chu IS Leem SH Minisatellite polymorphisms of the SLC6A19: susceptibility in hypertension Biochem Biophys Res Commun 2008 374 4 714 719 10.1016/j.bbrc.2008.07.094 18671945

[B12] Paquette J Giannoukakis N Polychronakos C Vafiadis P Deal C The INS 5' variable number of tandem repeats is associated with IGF2 expression in humans J Biol Chem 1998 273 23 14158 14164 10.1074/jbc.273.23.14158 9603916

[B13] Fuke S Suo S Takahashi N Koike H Sasagawa N Ishiura S The VNTR polymorphism of the human dopamine transporter (DAT1) gene affects gene expression Pharmacogenomics J 2001 1 2 152 156 11911442 10.1038/sj.tpj.6500026

[B14] Wu KJ Grandori C Amacker M Simon-Vermot N Polack A Lingner J Dalla-Favera R Direct activation of TERT transcription by c-MYC Nat Genet 1999 21 2 220 224 10.1038/6010 9988278

[B15] Horikawa I Cable PL Mazur SJ Appella E Afshari CA Barrett JC Downstream E-box-mediated regulation of the human telomerase reverse transcriptase (hTERT) gene transcription: evidence for an endogenous mechanism of transcriptional repression Mol Biol Cell 2002 13 8 2585 2597 10.1091/mbc.E01-11-0107 12181331 PMC117927

[B16] Chakravarti A Lynn A Birren B, Green ED, Klapholz S, Myers RM, Roskams Meiotic mapping in human Genome analysis: A laboratory manual 1999 New York: Cold Spring Harbor Laboratory Press 1 69

[B17] Song C Kang T Ro JY Lee MS Kim CS Ahn H Nomograms for the prediction of pathologic stage of clinically localized prostate cancer in Korean men J Korean Med Sci 2005 20 2 262 266 10.3346/jkms.2005.20.2.262 15831998 PMC2808603

[B18] Kang T Song C Song GH Shin GH Shin DI Kim CS Ahn H The anatomic distribution and pathological characteristics of prostate cancer: A mapping analysis Korean J Urol 2006 47 6 578 585

[B19] Lee YJ Kim DI Lee HE Won JK Hong EK Lee GK Lee KH Park WS Pathologic features of Korean prostate adenocarcinoma: Mapping analysis of 83 cases The Korean Journal of Pathology 2006 40 3 204 209

[B20] Greene KL Albertsen PC Babaian RJ Carter HB Gann PH Han M Kuban DA Sartor AO Stanford JL Zietman A Carroll P Prostate specific antigen best practice statement: 2009 update J Urol 2009 182 5 2232 2241 10.1016/j.juro.2009.07.093 19781717

[B21] Sinecen M Makinaci M Classification of prostate cell nuclei using artificial neural network methods Engineering and Technology 2005 7 170 172

[B22] Meigs JB Dupuis J Herbert AG Liu C Wilson PW Cupples LA The insulin gene variable number tandem repeat and risk of type 2 diabetes in a population-based sample of families and unrelated men and women J Clin Endocrinol Metab 2005 90 2 1137 1143 10.1210/jc.2004-1212 15562019

[B23] Wang Y Hu Z Liang J Wang Z Tang J Wang S Wang X Qin J Wang X Shen H A tandem repeat of human telomerase reverse transcriptase (hTERT) and risk of breast cancer development and metastasis in Chinese women Carcinogenesis 2008 29 6 1197 1201 10.1093/carcin/bgn099 18413362

[B24] Rosell R Calvo R Sánchez JJ Maurel J Guillot M Monzó M Núñez L Barnadas A Genetic susceptibility associated with rare HRAS1 varaiable number of tandem repeats alleles in Spanish non-small cell lung cancer patients Clin Cancer Res 1999 5 7 1849 1854 10430091

[B25] Weitzel JN Ding S Larson GP Nelson RA Goodman A Grendys EC Ball HG Krontiris TG The HRAS1 minisatellite locus and risk of ovalian cancer Cancer res 2000 60 2 259 61 10667571

[B26] Green M Krontiris TG Allelic variation of reporter gene activation by the HRAS1 minisatellite Genomics 1993 17 2 429 434 10.1006/geno.1993.1343 8406494

[B27] Turri MG Cuin KA Porter AC Characterisation of a novel minisatellite that provides multiple splice donor sites in an interferon-induced transcript Nucleic Acids Res 1995 23 11 1854 1861 10.1093/nar/23.11.1854 7596809 PMC306954

[B28] Ahn EK Kim WJ Kwon JA Choi PJ Kim WJ Sunwoo Y Heo J Leem S Variants of MUC5B minisatellites and the susceptibility of bladder cancer DNA Cell Biol 2009 28 4 169 176 10.1089/dna.2008.0827 19191526

[B29] Colgin LM Reddel RR Telomere maintenance mechanisms and cellular immortalization Curr Opin Genet Dev 1999 9 1 97 103 10.1016/S0959-437X(99)80014-8 10072358

[B30] Horikawa I Barrett JC Transcriptional regulation of the telomerase hTERT gene as a target for cellular and viral oncogenic mechanisms Carcinogenesis 2003 24 7 1167 1176 10.1093/carcin/bgg085 12807729

[B31] Meyerson M Role of telomerase in normal and cancer cells J Clin Oncol 2000 18 13 2626 2634 10893296 10.1200/JCO.2000.18.13.2626

[B32] Shah J Khaksar SJ Sooriakumara P Management of prostate cancer. Part 3: metastatic disease Expert Rev Anticancer Ther 2006 6 5 813 821 10.1586/14737140.6.5.813 16759171

[B33] Onda M Li D Suzuki S Nakamura I Takenoshita S Brogren CH Stampanoni S Rampino N Expansion of microsatellite in the thyroid hormone receptor-alpha1 gene linked to increased receptor expression and less aggressive thyroid cancer Clin Cancer Res 2002 8 9 2870 2874 12231529

[B34] Suh J Rabson AB NF-kappaB activation in human prostate cancer: important mediator or epiphenomenon? J Cell Biochem 2004 91 1 100 117 10.1002/jcb.10729 14689584

[B35] Ye Q Chung LW Li S Zhau HE Identification of a novel FAS/ER-alpha fusion transcript expressed in human cancer cells Biochim Biophys Acta 2000 1493 3 373 377 11018265 10.1016/s0167-4781(00)00202-5

[B36] Brandt B Meyer-Staeckling S Schmidt H Agelopoulos K Buerger H Mechanisms of *egfr *Gene Transcription Modulation: Relationship to Cancer Risk and Therapy Response Clin Cancer Res 2006 12 24 7252 7260 10.1158/1078-0432.CCR-06-0626 17189396

